# FTY720 inhibits proliferation and epithelial-mesenchymal transition in cholangiocarcinoma by inactivating STAT3 signaling

**DOI:** 10.1186/1471-2407-14-783

**Published:** 2014-10-25

**Authors:** Zhaoyang Lu, Jiabei Wang, Tongsen Zheng, Yingjian Liang, Dalong Yin, Ruipeng Song, Tiemin Pei, Shangha Pan, Hongchi Jiang, Lianxin Liu

**Affiliations:** Department of Hepatic Surgery, The First Affiliated Hospital of Harbin Medical University, Key Laboratory of Hepatosplenic Surgery, Ministry of Education, No. 23 Youzheng Street, Harbin, Heilongjiang Province 150001 China; Department of Pharmacology (the State-Province Key Laboratories of Biomedicine-Pharmaceutics of China, Key Laboratory of Cardiovascular Research, Ministry of Education), Harbin Medical University, Harbin, China

**Keywords:** Cholangiocarcinoma, FTY720, STAT3, Apoptosis, Cell cycle

## Abstract

**Background:**

Interleukin 6 (IL-6)-mediated signal transducers and activators of transcription 3 (STAT-3) phosphorylation (activation) is aberrantly sustained in cholangiocarcinoma cells resulting in enhanced myeloid cell leukemia 1 (Mcl-1) expression and resistance to apoptosis. FTY720, a new immunosuppressant, derived from ISP-1, has been studied for its putative anti-cancer properties. This study aimed to elucidate the mechanism by which FTY720 mediates antitumor effects in cholangiocarcinoma (CC) cells.

**Methods:**

Three CC cell lines were examined, QBC939, TFK-1, and HuCCT1. The therapeutic effects of FTY720 were evaluated *in vitro* and *in vivo*. Cell proliferation, apoptosis, cell cycle, invasive potential, and epithelial- mesenchy-mal transition (EMT) were examined.

**Results:**

FTY720 greatly inhibited CC cells proliferation and EMT *in vitro* and *in vivo*, and this effect was associated with dephosphorylation of STAT3^tyr705^. FTY720 induced apoptosis and G1 phase arrest in CC cells, and inhibited invasion of CC cells. Western blot analysis showed that FTY720 induced cleavage of caspases 3, 8 and 9, and of PARP, in a dose-dependent manner, consistent with a substantial decrease in p-STAT3, Bcl-xL, Bcl-2, survivin, cyclin D1, cyclin E, N-cadherin, vimentin, VEGF and TWIST1. *In vivo* studies showed that tumor growth and metastasis were significantly suppressed after FTY720 treatment.

**Conclusions:**

These results suggest that FTY720 induces a significant decrease in p-STAT3, which inhibits proliferation and EMT of CC cells, and then induces G1 phase arrest and apoptosis. We have characterized a novel immunosuppressant, which shows potential anti-tumor effects on CC via p-STAT3 inhibition. FTY720 merits further investigation and warrants clinical evaluation.

**Electronic supplementary material:**

The online version of this article (doi:10.1186/1471-2407-14-783) contains supplementary material, which is available to authorized users.

## Background

Human cholangiocarcinoma (CC) arises from the epithelium of the biliary tree. CC encompasses adenocarcinomas arising in the intra or extrahepatic biliary tree and in the gall bladder. CC is a relatively uncommon malignancy in western countries [1], but has a high incidence in Asia and Latin America [2, 3]. CC is characterized by poor prognosis and a 5-year survival rate less than 5% [4]. Currently, conventional chemotherapy and radiotherapy have not been reported to be effective in improving long-term survival [5], the only curative treatment for CC is surgical resection. However, the majority of CC patients shows advanced liver involvement and metastasis, and this precludes the use of curative surgical resection. Therefore, there is an urgent need to define the molecular mechanisms underlying CC proliferation and metastasis in order to develop novel therapeutic strategies.

One promising candidate for CC targeted therapy is signal transducer and activator of transcription 3 (STAT3). STAT3 is a transcription factor that is constitutively activated in many types of cancer, contributing to tumor progression via several mechanisms. [6–9] When phosphorylated at tyrosine^705^, STAT3 undergoes translocation from the cytosol to the nucleus, where it functions as a pivotal transcription factor upregulating gene transcription [10–12]. IL-6 secretion can further increase STAT3 activation levels within tumor cells via an autocrine feedback loop [6]. IL-6–activated STAT3 is crucial for survival of several types of cancer cell, including multiple myeloma, a plasmacytic B-cell malignancy [6, 13]. Studies suggest that IL-6/STAT3 signaling is aberrant in human CC cells and CC tissues with prolonged and sustained STAT-3 phosphorylation [14, 15]. The mechanisms responsible for this atypical IL-6 signaling response are unclear but of pathophysiological importance.

FTY720 is a synthetic sphingosine immunosuppressant, which is currently undergoing clinical trials for the prevention of kidney graft rejection [16] and the treatment of relapsing multiple sclerosis [17]. Previous studies indicate that the effect of FTY720 on prolonging the survival of allografts is attributable to the ability of its phosphorylated metabolite to inhibit T-lymphocyte infiltration by targeting several of the sphingosine-1-phosphate (S1P) receptors [18, 19]. Recently, FTY720 has been reported to have a strong antitumor effect on breast cancer [20], bladder cancer [21] and leukemia [22]. So far, the feasibility of using this drug in CC treatment has not been studied. The precise mechanism of FTY720 action on cancer cells is not completely understood. Therefore, in this study we aimed to investigate the *in vitro* and *in vivo* anticancer potential of FTY720 and to ascertain the precise mechanism by which proliferation and metastasis are inhibited in CC cells.

We investigated the effect of FTY720 on the STAT3 cell survival pathway and found that STAT3 dephosphorylation plays a central role in cell growth arrest, apoptosis and metastasis upon administration of FTY720 to CC cell lines. Dephosphorylation of STAT3^tyr705^ results in G1 arrest and apoptosis possibly by up-regulation of p27, cleavage of caspase-3 and down-regulation of Mcl-1, cyclin D1 and Bcl-xL. It might also inhibit EMT by up-regulation of E-cadherin and down-regulation of Vimentin and N-cadherin, both in vitro and in vivo.

## Methods

### Cell lines and reagents

The human CC cell line QBC939 was a gift from Prof. Shuguang Wang (Third Military Medical University, Chongqing, China). Human CC cell lines TFK-1 and HuCCT1 were kindly provided by the Cancer Cell Repository, Tohoku University, Japan. All cell lines were cultured in Dulbecco’s modified Eagle's medium (DMEM; Gibco BRL, Grand Island, NY, USA) supplemented with 10% fetal bovine serum (Gibco BRL), penicillin G (100,000 U/L) and streptomycin (100 mg/L; Gibco BRL) at 37°C in a humidified atmosphere containing 5% CO_2_. FTY720 was purchased from Selleckchem (Houston, TX, USA).

### MTT assay

Cell viability was assessed using the MTT assay. CC cells were seeded at 2 × 10^4^ per well in 96-well flat-bottomed plates and incubated in 10% FBS supplemented DMEM for 24 h. Cells were treated with FTY720 at various concentrations in the same medium. Controls received dimethyl sulfoxide (DMSO) vehicle at a concentration equal to that in drug-treated cells. After 24 and 48 h, the drug-containing medium was replaced with 200 μL of 10% FBS supplemented DMEM containing 0.5 mg/mL MTT, and cells were incubated in the CO_2_ incubator at 37°C for 4 h. Medium was removed and the reduced MTT solubilized in 100 μL per well of DMSO. Absorbance was then measured at 570 nm. Six replicates were performed for each experiment.

### Cell cycle analysis

Cells were treated with FTY720 and then 10^6^ cells were fixed in 80% ethanol at -20°C for 24 h. Fixed cells were stained according to the Cycle TESTTM PLUS DNA Reagent Kit protocol (BD Biosciences, San Jose, CA, USA) and analyzed by flow cytometry (Beckman Coulter FC 500). The experiment was repeated thrice under the same conditions.

### Apoptosis analysis

FTY720 treated cells were harvested, washed twice with prechilled PBS and resuspended in 1× binding buffer at a concentration of 1 × 10^6^ cells/ml. One hundred microliters of this cell suspension (1 × 10^5^ cells) was mixed with 5 μl of Annexin V-FITC and 5 μl of propidium iodide (PI) (BD Biosciences) according to the manufacturer’s instructions. The mixed solution was gently vortexed and incubated in the dark at room temperature (25°C) for 15 min. Four hundred microliters of 1× dilution buffer were then added to each tube and cell apoptosis analysis was performed by flow cytometry (BD FACS Calibur) within 1 h.

### Cell invasion assays

Eight hours after FTY720 treatment, invasion was measured using 24-well BioCoat cell culture inserts (BD Biosciences, NJ, USA) with an 8 μm porosity polyethylene terephthalate membrane coated with Matrigel Basement Membrane Matrix.

### Tumor xenografts in nude mice

In these studies, tumor xenografts were established by standard techniques in 8-week-old nude mice (BALBc nu/nu) [23]. In brief, each mouse was injected subcutaneously with 3 × 10^6^ QBC939 cells and 3 × 10^6^ HuCCT1 cells suspended in PBS. Tumor size was measured by Vernier calipers, and tumor volume was calculated as described previously [24]. Once the tumors reached an average of 90 mm^3^, the treatment began. For the treatment group, FTY720 was administered by daily i.p. injection of 10 mg/kg/day for 20 days. After treatment, mice in both the treatment and control groups (n = 10 in each group) were sacrificed. Tumor tissues were collected, snap-frozen and embedded in paraffin for further analysis.

### Ethics statement

This study does not involve human subjects, human material, or human data. All nude mice were treated and all procedures were conducted in accordance with the guidelines for experimental animals approved by the Animal Care and Use Committee of Harbin Medical University, Harbin, China.

### *In vivo*invasive assay

HuCCT1 cells (3 × 10^6^ cells in 200 μL) and QBC939 (3 × 10^6^ cells in 200 μL) were injected into the intraperitoneal cavity as previously described [25]. Animals were randomized to receive either FTY720 (10 mg/kg/d, i.p.) or vehicle at 1 week after injection. The mice were sacrificed at 4 weeks after tumor cell injection.

### Western blot analysis

Protein isolation was performed as described previously [26], and western blot analysis was achieved via established protocols [27]. The primary antibodies used were against N-cadherin, E-cadherin, p16 and vimentin (Abcam, Cambridge, MA, USA); p27, STST3, p-STAT3, cleaved PARP, cleaved caspase-3, cleaved caspase-8, cleaved caspase-9, Bcl-xL, and Bcl-2 (Cell Signaling Technology, Danvers, MA, USA); cyclin D1, VEGF, TWIST1, Bax, survivin, cyclin E, CDK2, CDK4 and β-actin (Santa Cruz Biotechnology, Santa Cruz, CA, USA).

### Immunofluorescence

Briefly, cells seeded on coverslips were fixed with 4% (w/v) paraformaldehyde (Sigma-Aldrich) for 10 min and permeabilized with 0.1% (v/v) Triton X-100 for 5 min at room temperature. The cells were then incubated overnight with primary antibodies at 4°C, followed by incubation with fluorescent secondary antibody for 1 h at room temperature. After final washes with PBS, coverslips were mounted using an anti-fade mounting solution containing 4',6-diamidino-2-phenylindole (DAPI; Vector Lab) and images were examined and captured.

### Immunohistochemistry

Immunohistochemistry was performed as described previously [28] using Ki-67, CD31 and cleaved caspase-3 antibodies (Cell Signaling Technology).

### Statistical analysis

All data are expressed as mean values ± standard deviation (SD). Comparisons among multiple groups were made with a one-way analysis of variance followed by Dunnett's t-test. A value of “*p* <0.05” was considered to be statistically significant.

## Results

### FTY720 is a potent anti-CC agent and induces apoptosis in CC cells

**Figure 1:**
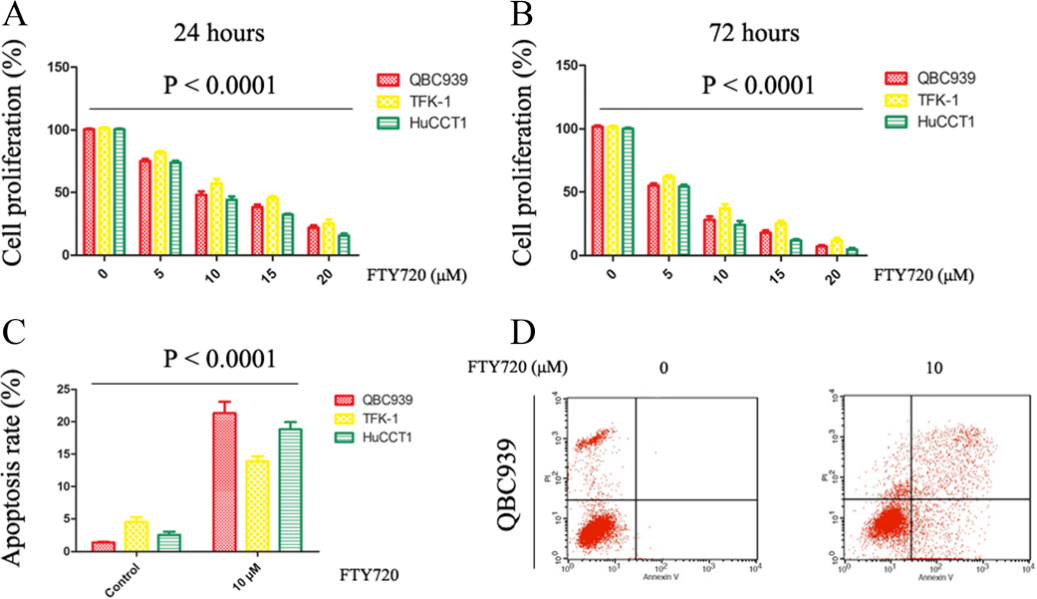
**FTY720 is cytotoxic to CC cells in a dose- and time-dependent manner. (A)** MTT assay showing percentage of viable CC cells treated with 0, 5, 10, 15 and 20 μmol/L of FTY720 for 24 h. Data are presented as mean ± SD from three independent experiments. **(B)** CC cells were treated with FTY720 or vehicle for 72 hr, and proliferation measured using MTT assays. **(C)** Flow cytometry results of annexin V-PI stained CC cells after exposure to FTY720 (0 or 10 μmol/L) for 24 h. An increase in apoptotic cells following treatment with FTY720 is shown. Data are presented as the mean ± SD from three independent experiments. **(D)** A representative example of apoptosis of QBC939 cells treated with 10 μmol/L of FTY720 for 24 h.

The *in vitro* activity of FTY720 against CC cells was evaluated after 24 h of exposure to drug. Cells were grown in the absence or presence of different concentrations (0, 5, 10, 15 and 20 μmol/L) of FTY720, and cytotoxicity was measured by the MTT assay. FTY720 effectively induced cell death in all cell lines tested (Figure 1A). The IC_50_ of FTY720 after 24 h of exposure to the drug was 9.81, 11.66 and 8.84 μmol/L for QBC939, TFK-1 and HuCCT1 cells, respectively. Extending drug exposure to 72 h resulted in additional cytotoxicity, indicating that FTY720 also induced cell death in a time-dependent manner (Figure 1B).To determine whether CC cell death induced by FTY720 involves apoptosis, flow cytometric analysis with annexin V–PI staining was performed. FTY720 induced obvious apoptosis in all cell lines tested at the dose of 10 μmol/L after 24 h (Figure 1C). Figure 1D is a representative example of apoptosis of QBC939 cells treated with 10 μmol/L FTY720 for 24 h.

### FTY720 induces cell death in a caspase-dependent manner by cleavage of caspases 3, 8 and 9

**Figure 2:**
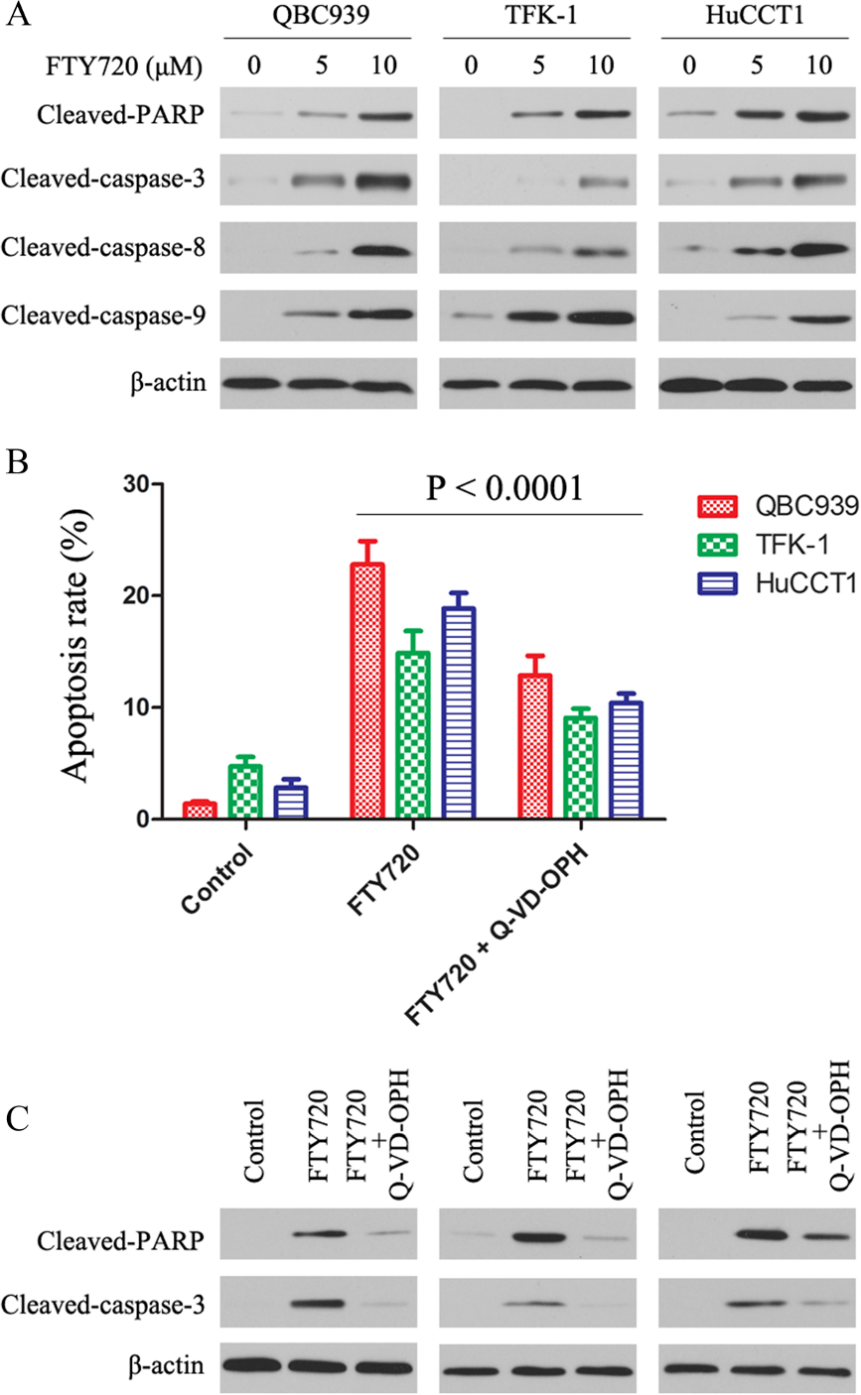
**FTY720 induces cell death in a caspase-dependent manner. (A)** Caspase activation and PARP cleavage following treatment with FTY720. Lysates from CC cells treated with 0, 5 and 10 μmol/L FTY720 for 24 h were probed for cleaved-caspase-3, cleaved-caspase-8, cleaved-caspase-9 and cleaved-PARP by western blotting. **(B)** Caspase inhibition protects against FTY720-induced cell death. CC cells were incubated in 0.1% DMSO, 5 μmol/L FTY720 or a combination of Q-VD-OPH (20 μmol/L) and 5 μmol/L FTY720, followed by annexin V-PI staining 24 h later. Data are presented as the mean ± SD from three independent experiments. **(C)** Lysates from treated cells were probed for cleaved-caspase-3 or cleaved-PARP by western blotting. The western blot is representative of three independent experiments. β-Actin was used as the internal control.

Next, we explored the effect of FTY720 on caspase-dependent apoptotic pathways. FTY720 induced cleavage of caspases 3, 8 and 9, and of PARP, in a dose-dependent manner after 24 h incubation with the drug (Figure 2A). To determine the dependence of FTY720-induced apoptosis on the caspase pathway, we assessed the ability of the pan-caspase inhibitor, Q-VD-OPH to protect against cell death. As shown in Figure 2B, Q-VD-OPH reduced FTY720-induced cell death as determined by annexin V-PI staining and the effect was only partial. We next examined whether Q-VD-OPH actually inhibited FTY720 activation of caspase-3 as measured by processing of the proform and downstream cleavage of PARP, which is characteristic of caspase-dependent apoptosis. CC cells were exposed to FTY720 in the presence or absence of Q-VD-OPH and cell lysates made. As shown in Figure 2C, Q-VD-OPH greatly diminished PARP and caspase-3 cleavage as well as preventing cell death. Together, these data demonstrate that while apoptosis is induced by FTY720 mainly through caspase-dependent mechanisms, non-caspase dependent pathways may also operate.

### FTY720 inhibits constitutive and inducible STAT3 phosphorylation in CC cells, and affects the expression of anti- or proapoptotic proteins

**Figure 3:**
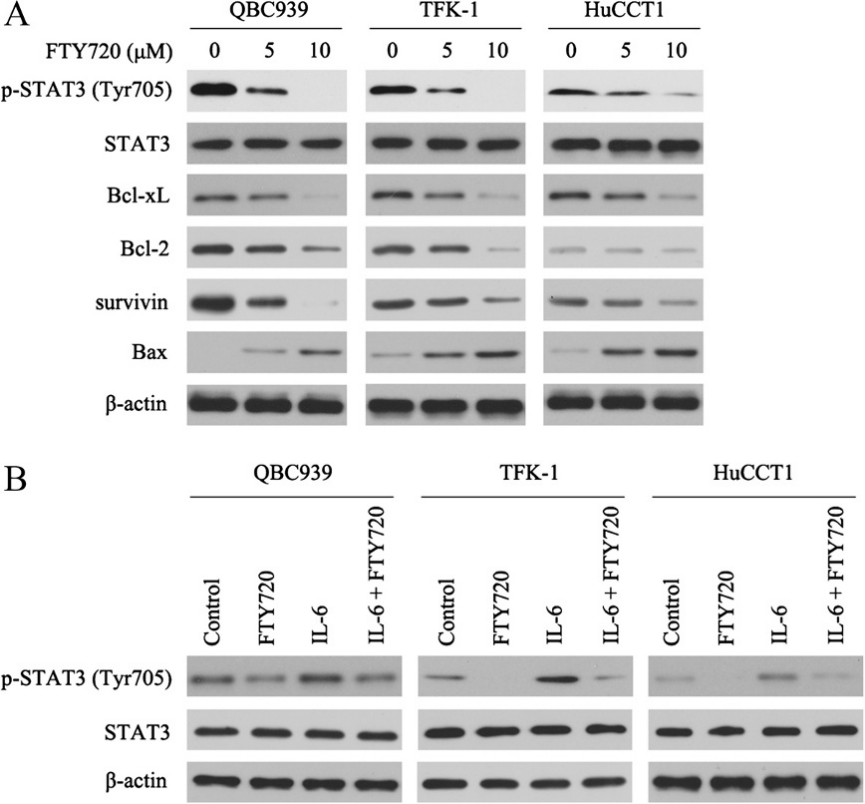
**FTY720 reduces constitutive and inducible p-STAT3 in CC cells, and downregulates the expression of anti- or proapoptotic proteins. (A)** CC cells were treated for 24 h with or without FTY720 and analyzed for the indicated protein by western blotting. **(B)** FTY720 reduced IL-6 induced p-STAT3 expression in CC cells as shown in the western blot. β-Actin was used as the internal control. All assays were performed in triplicate.

We first evaluated the effect of FTY720 on the expression of p-STAT3 in CC cells. Figure 3A shows that treatment of CC cells with FTY720 for 24 h significantly reduced the level of in tyrosine-phosphorylated STAT3 although total STAT3 was unaffected. FTY720 treatment also strongly decreased the expression of Bcl-xL, Bcl-2, survivin and increased the expression of Bax in CC cells. Next, we examined whether FTY720 could inhibit IL-6-induced STAT3 phosphorylation in CC cells. CC cells were pretreated with FTY720 (5 μM) for 24 h and then stimulated with IL-6 (10 ng/ml) for 15 min. As shown in Figure 3B, IL-6 induced STAT3 phosphorylation was reduced by FTY720. These results indicate that the STAT3 pathway is likely to be an important target of FTY720 in CC cells.

### FTY720 downregulates cyclin D1 and cyclin E, increases p27 and p16 expression and induces G1 cell cycle arrest in CC cells

**Figure 4:**
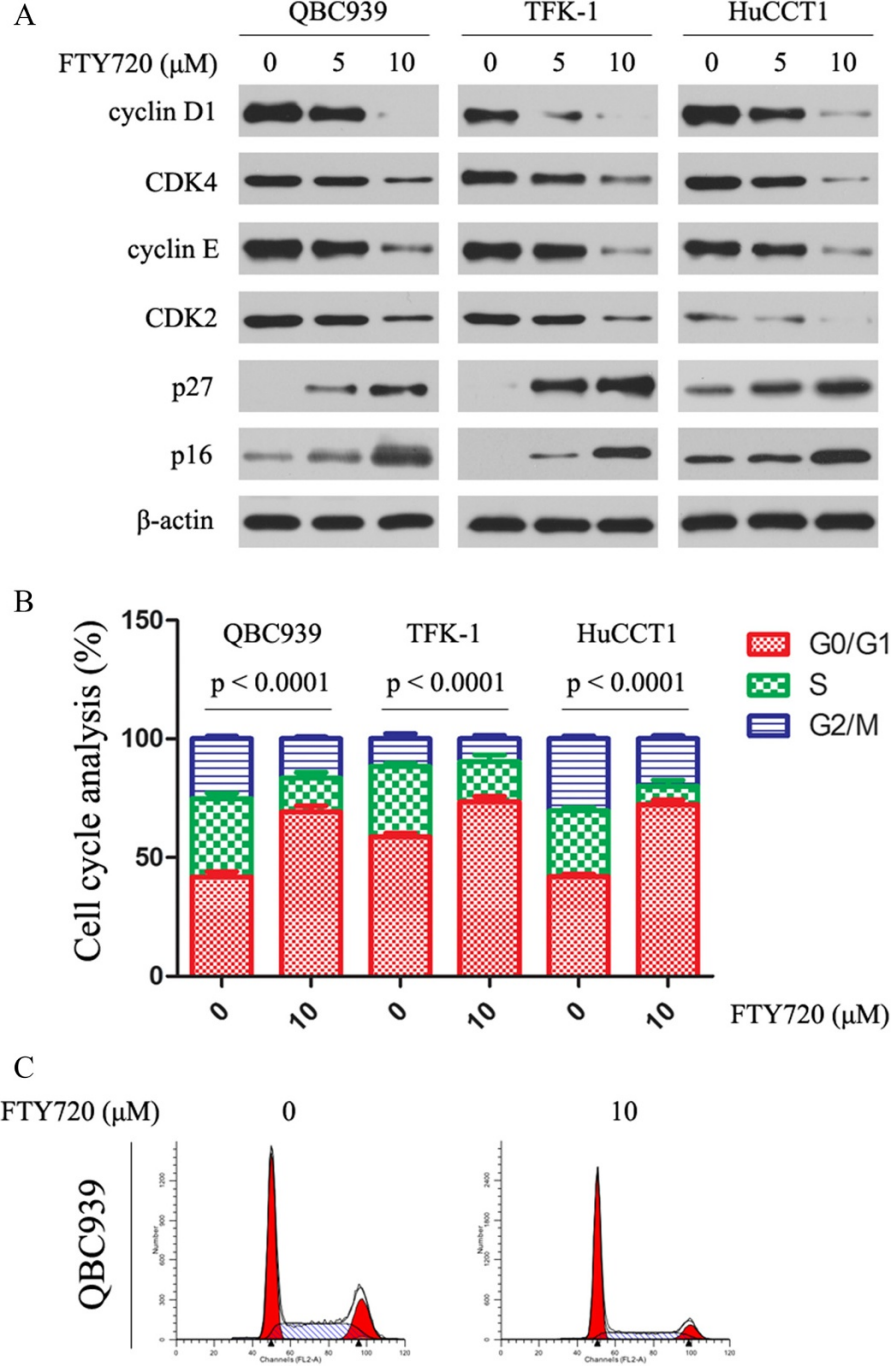
**Effect of FTY720 on cell cycle proteins and cell cycle progression. (A)** FTY720 induces expression of p16 and p27 and reduces expression of cyclin D1, CDK4, cyclin E and CDK2. CC cells were treated with FTY720 at the indicated concentrations for 24 h. Lysates were then prepared immediately and analyzed by western blotting for cyclin D1, CDK4, cyclin E, CDK2, p16 and p27. β-Actin was used as the internal control. All assays were done in triplicate. **(B)** Cell cycle analysis of FTY720-treated CC cells showing arrest in G1 phase. CC cells were incubated with FTY720 for 24 h. The percentage of cells in each phase of the cell cycle is presented as the mean ± SD from three independent experiments. Following treatment with FTY720 for 24 h, there was a significant increase in the percentage of cells in G0/G1 relative to the control group. **(C)** A representative example of cell cycle arrest in QBC939 cells treated with FTY720 for 24 h.

Next, we investigated the effect of FTY720 on cell cycle arrest of CC cells. As shown in Figure 4A, reductions in the levels of cyclin D1, CDK4, cyclin E and CDK2 were observed after FTY720 treatment for 24 h. As increased expression of p27 results in inhibition of proliferation, we examined the effect of FTY720 on its expression and on that of p16, another cell cycle inhibitor that has been shown to be transcriptionally silenced in CC [29]. Expression of both p27 and p16 proteins was induced by FTY720 after treatment for 24 h (Figure 4A). Consistent with the above findings, cell cycle analysis showed that FTY720 induces G1 cell cycle arrest in CC cells (Figure 4B, C).

### FTY720 inhibits the invasive potential of CC cells in vitro

**Figure 5:**
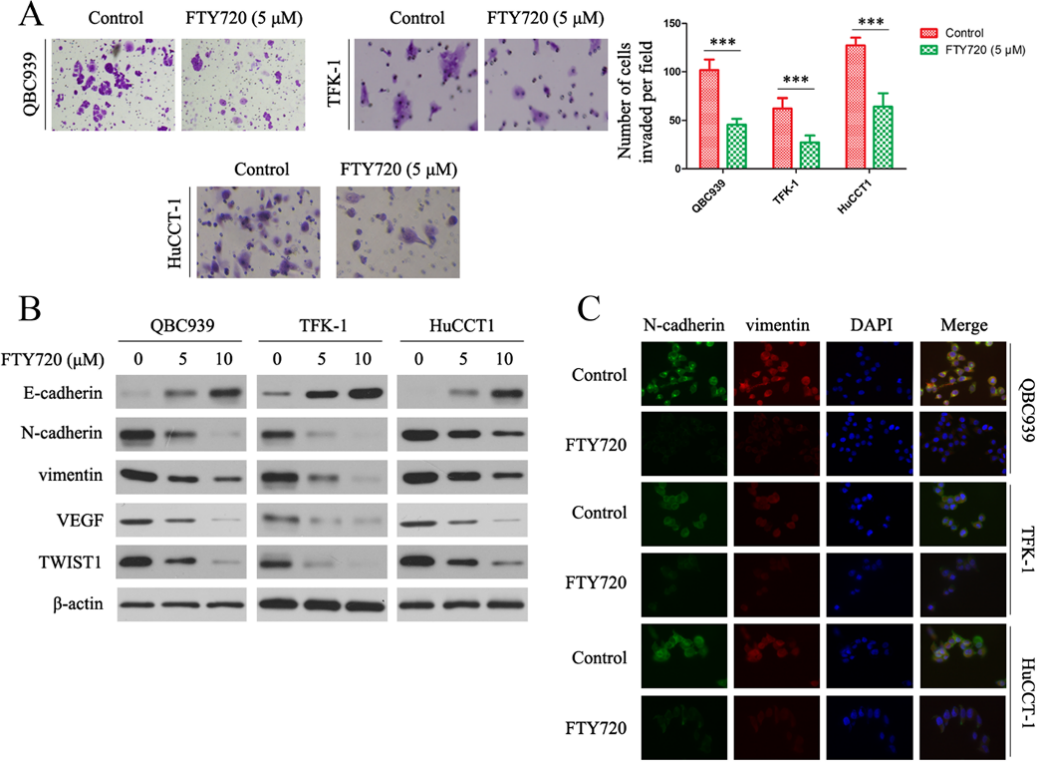
**FTY720 inhibits the invasive potential of CC cells** ***in vitro*** **. (A)** FTY720 significantly inhibited the invasive capacity of QBC939, TFK-1 and HuCCT1 cells. ***P <0.0001. The results are presented as the mean ± SD of experiments performed in triplicate. **(B)** Western blot showing that FTY720 increased the expression of E-cadherin and decreased the expression of N-cadherin, vimentin, VEGF and TWIST1 in CC cells. β-Actin was used as the internal control. All assays were performed in triplicate. **(C)** Single and merged images show immunofluorescence staining of N-cadherin (green) and vimentin (red). The cell nucleus is stained blue by DAPI.

To determine the function of FTY720, we treated QBC939, TFK-1 and HuCCT1 cells with FTY720. FTY720 significantly inhibited their invasive capacity, as compared with DMSO-treated cells (Figure 5A). Given that FTY720 inhibits CC invasion, we investigated the effect of FTY720 on epithelial-mesenchymal transition (EMT), a critical event in tumor invasion. Western blot analysis indicated a higher expression of E-cadherin in CC cells treated with FTY720. In contrast, the expression of N-cadherin, vimentin, VEGF and TWIST1 decreased in FTY720 treated CC cells (Figure 5B). As shown by immunofluorescence (Figure 5C), FTY720 markedly reduced N-cadherin and vimentin levels in CC cells, which was in good agreement with the results in Figure 5B.

### *FTY720 inhibits tumor growth and metastasis of CC*in vivo

**Figure 6:**
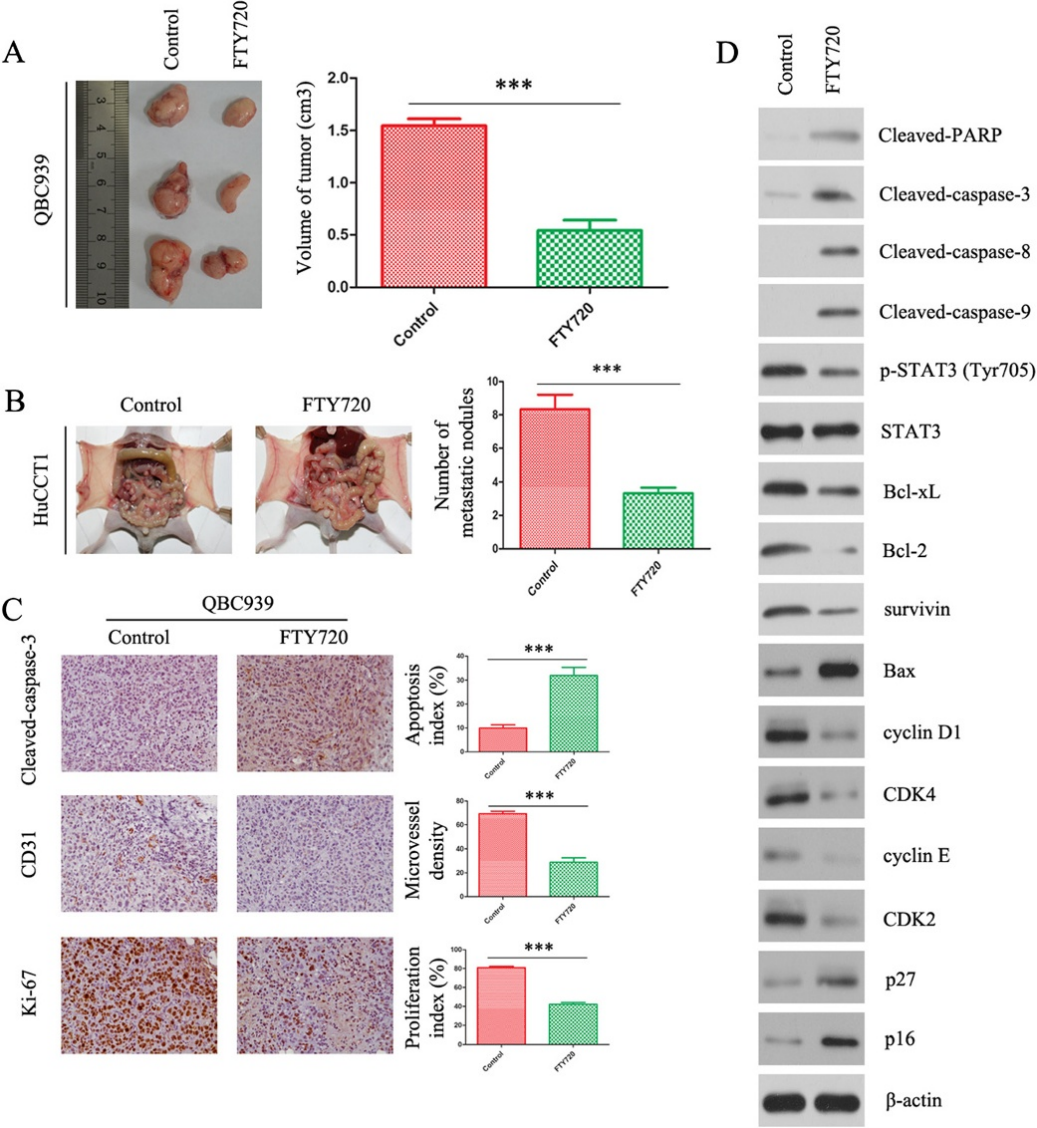
**FTY720 inhibits proliferation and metastasis of CC** ***in vivo*** **. (A)** Photomicrographs of xenograft tumors in nude mice. Representative images of a mouse in each group are presented. Tumor volumes in FTY720-treated mice were smaller than those of control mice. **(B)** The multiple tumor masses formed by the HuCCT1 cells in the FTY720-treated group were much smaller than those formed by HuCCT1 cells in the control group. **(C)** Tumors from different groups were immunostained for indicated molecules. CD31-stained microvessels were counted to record microvessel density. Apoptotic cells were counted to give the apoptosis index and cells expressing Ki-67 were counted to calculate the proliferation index. Pictures are representative of three independent experiments. **(D)** Western blot detection of the indicated molecules in tumor samples. β-Actin was used as the internal control. All assays were performed in triplicate. The results are expressed as the mean ± SD of three independent experiments; ***P <0.0001.

We further examined the effect of FTY720 on CC growth by establishing a xenograft CC model in nude mice. QBC939 and HuCCT1 was used for *in vivo* studies. Compared with the control group, FTY720 treatment resulted in a significant decrease of tumor size (Figure 6A and Additional file 1: Figure S1). The effects of FTY720 on the metastatic phenotype of CC were also examined *in vivo* by implanting HuCCT1 and QBC939 cells into the peritoneal cavity of nude mice. Necropsy after 4 weeks revealed that the control cells extensively colonized the visceral organs and formed multiple metastatic nodules (Figure 6B and Additional file 2: Figure S2), while the number of metastatic nodules was reduced in FTY720-treated mice. In addition, the body weight of mice from treated group was similar to the control group (Additional file 3: Figure S3A and S3B), indicating FTY720 suppress CC growth and metastasis without notable toxic side-effects. Immunohistochemistry showed changes of Ki-67, cleaved-caspase-3 and CD31 in the different groups (Figure 6C) respectively assess tumors’ ability of proliferation, apoptosis, and forming microvessels. The relative levels of the above mentioned proteins were also analyzed in the different groups by western blotting (Figure 6D). Together, these results reveal a high propensity of FTY720 to inhibit proliferation and metastasis in CC.

## Discussion

Cholangiocarcinoma is an aggressive disease, with a poor response to the treatments that are currently available, including the standard gemcitabine [30, 31]. To this end, we examined a new agent for the treatment of CC. FTY720 is a chemical substance derived by modifying an immunosuppressive metabolite and has been shown to possess anti-cancer properties in various types of cancer [32]. However, the efficacy of FTY720 against CC has not been previously assessed. Herein, we have demonstrated that FTY720 induces apoptosis and cell cycle arrest, inhibits EMT of CC cells and *in vivo* tumor growth in a nude mouse model without notable toxic side-effects.

The IL-6/STAT3 pathway plays an important role in human cancers. STAT proteins comprise a seven member family of latent cytoplasmic transcription factors [10, 33]. Accumulating data suggest that aberrant STAT signaling, and in particular STAT3 initiated cascades, participate in the development and progression of human cancers [10, 11]. Numerous studies have shown that STAT3 inhibitors have tumor suppressive effects on various tumors. AG490, the most popular STAT3 inhibitor, can induce CC cell apoptosis and inhibit CC/mycosis fungoides tumor cell proliferation [14, 15, 34]. New STAT3 inhibitors also can inhibit tumor proliferation [35, 36], chemo-therapy resistance [37] and metastasis [38]. We therefore presumed that STAT3 would be a good target for CC treatment, and our results indeed show that FTY720 inhibited proliferation and EMT in CC mainly through the IL-6/STAT3 pathway.

FTY720 has been demonstrated to inhibit proliferation of various tumors [20, 39, 40]. So we tried to test whether FTY720 could inhibit CC proliferation. Cell viability analysis (MTT assays) showed that FTY720 could induce a dramatic reduction in cell viability in all three CC cell lines tested. After cells were treated with FTY720 for 24 h, we observed a significant decrease in the S-phase population, and induction of G1 arrest. FTY720 induced significant expression of the cyclin-dependent kinase (CDK) inhibitors p16 and p27. Both p16 and p27 block the formation of cyclin-CDK complexes, allowing Rb to become activated and to halt the cell cycle. In addition to inducing p16 and p27, FTY720 also downregulated cyclin D1 and cyclin E in CC cells contributing to arrest in the G1 phase.

FACS analysis also showed that the inhibitory effect on CC cell growth by FTY720 was also related to induction of apoptosis. Our results show that FTY720-induced apoptosis is associated with cleavage of caspases 8, 9 and 3, and PARP, suggesting that the drug activates both the extrinsic and intrinsic apoptotic pathways. Further, FTY720-induced apoptosis is in large part dependent on caspase activation. In CC cells, FTY720 also modulates the expression of the antiapoptotic proteins. Of the Bcl-2 family members, the expression of Bcl-xL, Bcl-2 and survivin was significantly reduced, while Bax expression was increased. In additional to inhibition of tumor proliferation, numerous studies demonstrated that FTY720 could inhibit tumor metastasis [20, 41, 42]. And our study also demonstrate that incubation of CC cells with FTY720 leads to the loss of N-cadherin and vimentin and to the accumulation of E-cadherin. Furthermore, FTY720 significantly inhibited the invasive capacity of CC cells. We also examined the ability of FTY720 to suppress the growth and metastasis of human CC cancer cell xenografts in nude mice. We found a significant reduction in relative tumor size and metastatic nodules in FTY720-treated animals compared with untreated controls. In addition, the suppression of proliferation by FTY720 was confirmed by decreased Ki-67 expression. Increased numbers of apoptotic cells and activated protein levels of apoptosis–related proteins, such as cleaved-PARP, cleaved-caspase-9, cleaved-caspase-8 and cleaved-caspase-3, were accompanied by decreased p-STAT3 expression in the FTY720-treated animals.

IL-6 plays an important role in the growth and survival of CC cells [43, 44]. Our results indicate that FTY720 exerts an important inhibitory effect on the IL-6 signal transduction pathway by inhibiting constitutive and inducible STAT3 phosphorylation. STAT3 directly and indirectly upregulates the expression of genes that are required for uncontrolled proliferation and invasion of tumor cells [45, 46]. In our study, the FTY720-induced reduction of Bcl-xL, Bcl-2, N-cadherin, vimentin, cyclin E and cyclin D1 may result, at least in part, through an inhibitory effect on the STAT3 pathway. Importantly, FTY720 overcomes the activation of p-STST3 which was induced by IL-6.

## Conclusions

In conclusion, our results show that the novel synthetic sphingosine immunosuppressant, FTY720, has potent activity against CC *in vitro* and *in vivo*. Its ability to target mainly the IL-6/STAT3 pathway and downstream anti-apoptotic, EMT and cell cycle proteins, suggest its viability as part of the therapeutic armamentarium for CC. Our results provide preclinical rationale for clinical development of FTY720 for the treatment of CC.

## Electronic supplementary material

Additional file 1: Figure S1: FTY720 inhibits proliferation of CC *in vivo*. Photomicrographs of xenograft tumors in nude mice. Representative images of a mouse in each group are presented. Tumor volumes in FTY720-treated mice were smaller than those of control mice. *P < 0.05. (JPEG 467 KB)

Additional file 2: Figure S2: FTY720 inhibits metastasis of CC *in vivo*. The multiple tumor masses formed by the QBC939 cells in the FTY720-treated group were much smaller than those formed by QBC939 cells in the control group. *P < 0.05. (JPEG 437 KB)

Additional file 3: Figure S3: The graph showed the body weight of the animals with tumor xenografts/without tumor xenografts in the control and treatment groups throughout the treatment period. (JPEG 1 MB)
