# Supplementary figures and images for: FTY720 inhibits proliferation and epithelial-mesenchymal transition in cholangiocarcinoma by inactivating STAT3 signaling
Source: BMC Cancer. 2014 Oct 25;14:783. doi: 10.1186/1471-2407-14-783 (PMC4221672; doi:10.1186/1471-2407-14-783)

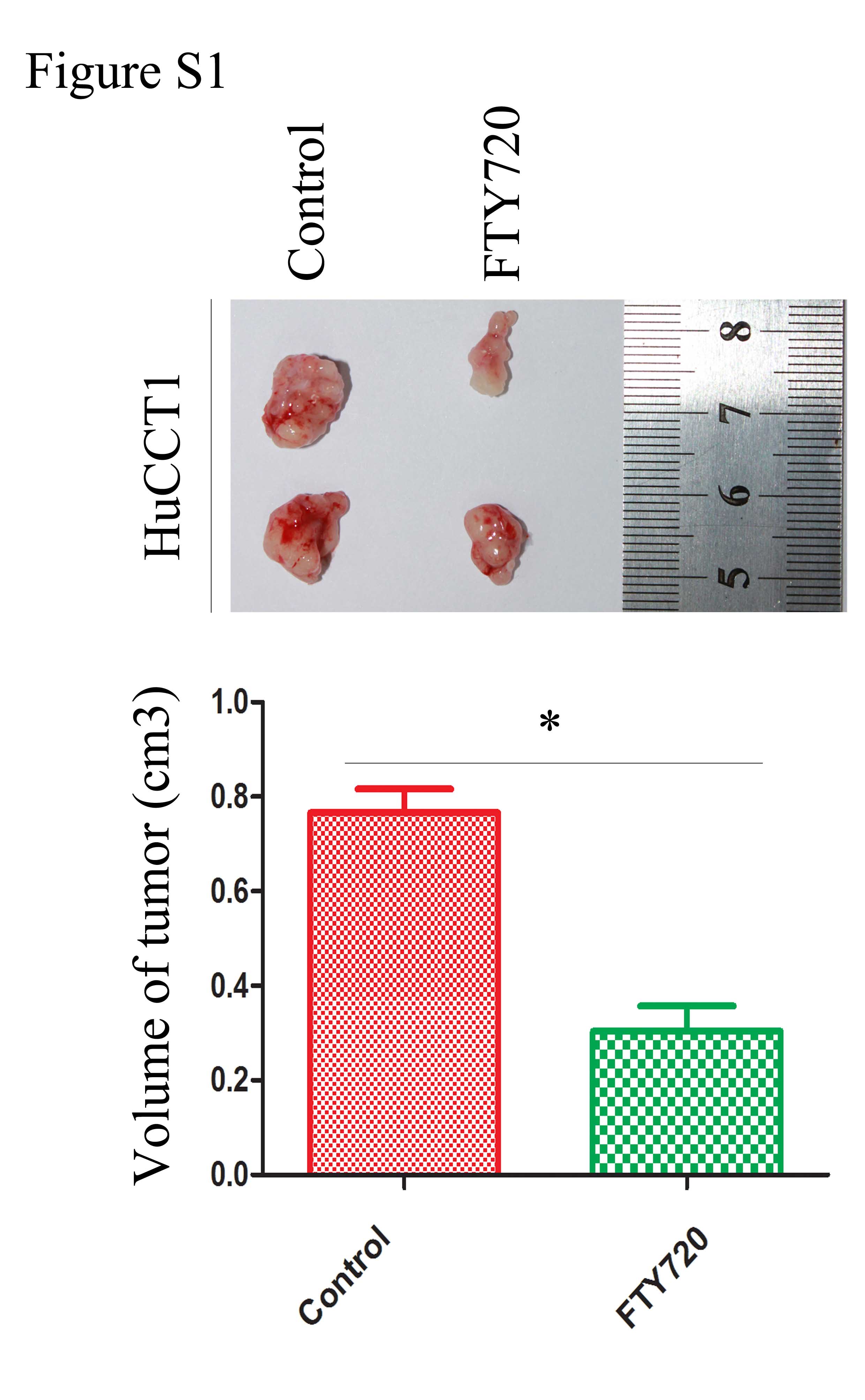

Supplement: Supplementary file 1 — Additional file 1: Figure S1: FTY720 inhibits proliferation of CC in vivo. Photomicrographs of xenograft tumors in nude mice. Representative images of a mouse in each group are presented. Tumor volumes in FTY720-treated mice were smaller than those of control mice. *P < 0.05. (JPEG 467 KB) [file 12885_2014_4964_MOESM1_ESM.jpeg]

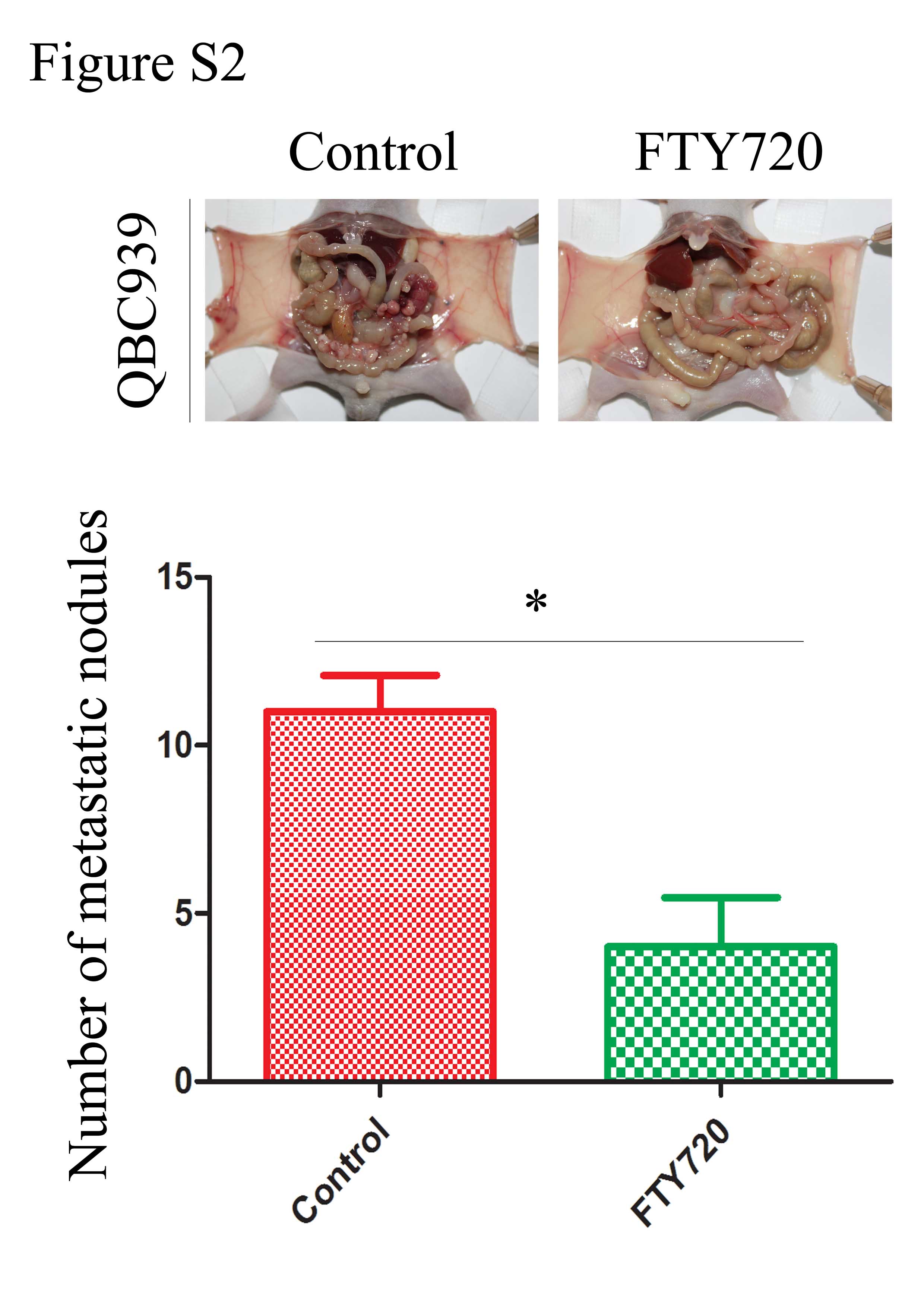

Supplement: Supplementary file 2 — Additional file 2: Figure S2: FTY720 inhibits metastasis of CC in vivo. The multiple tumor masses formed by the QBC939 cells in the FTY720-treated group were much smaller than those formed by QBC939 cells in the control group. *P < 0.05. (JPEG 437 KB) [file 12885_2014_4964_MOESM2_ESM.jpeg]

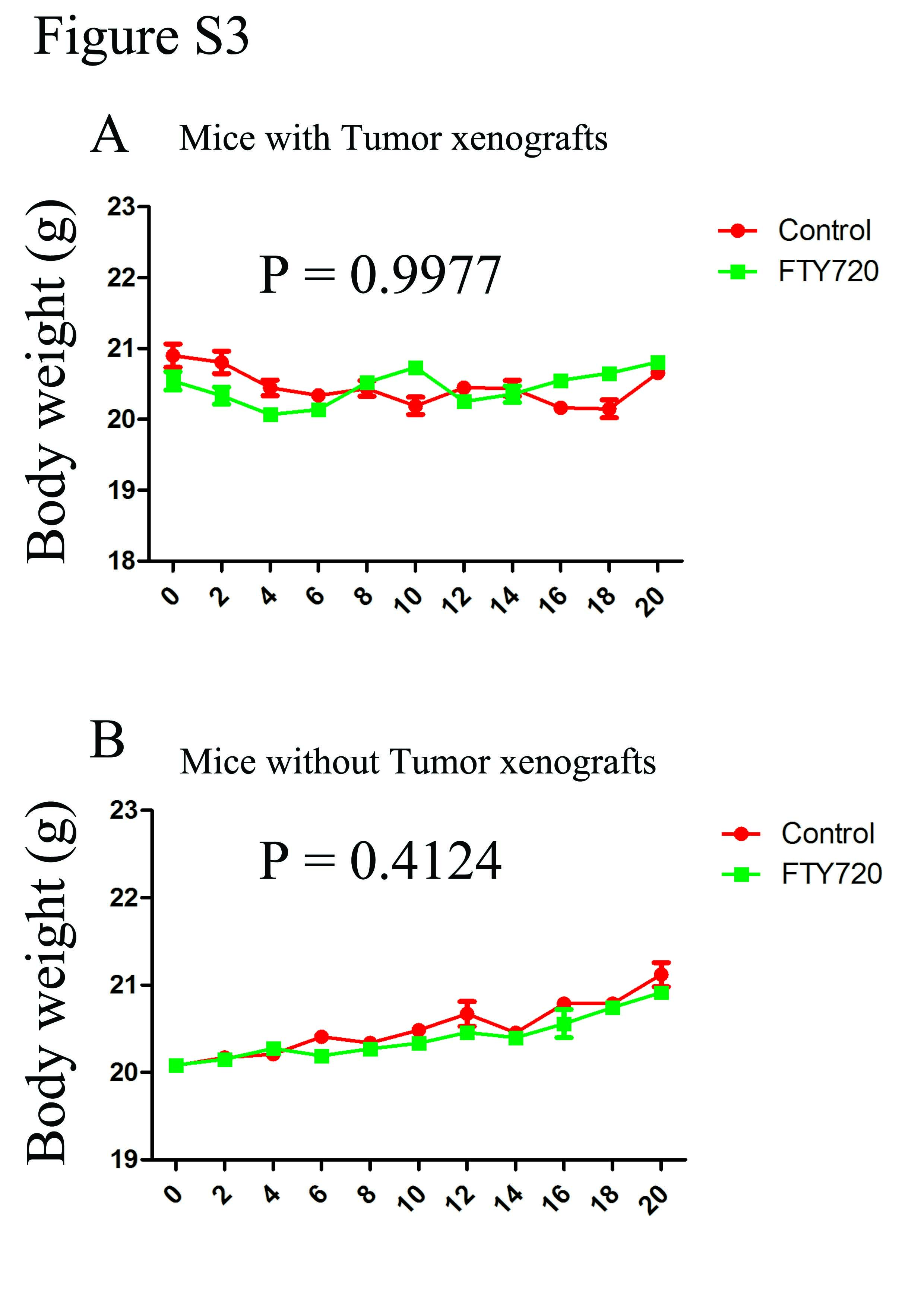

Supplement: Supplementary file 3 — Additional file 3: Figure S3: The graph showed the body weight of the animals with tumor xenografts/without tumor xenografts in the control and treatment groups throughout the treatment period. (JPEG 1 MB) [file 12885_2014_4964_MOESM3_ESM.jpeg]
